# The Hidden World within Plants

**DOI:** 10.3390/microorganisms10101951

**Published:** 2022-09-30

**Authors:** Philippe Jeandet, Rachid Lahlali, Essaid Ait Barka

**Affiliations:** 1 Research Unit Induced Resistance and Plant Bioprotection, USC INRAe 1488, SFR Condorcet FR CNRS 3417, Faculty of Sciences, University of Reims Champagne-Ardenne, 51687 Reims, France; 2 Phytopathology Unit, Department of Plant Protection, Ecole Nationale d’Agriculture de Meknès, Km 10, Rte Haj Kaddour, BP S/40, Meknes 50001, Morocco

Plants offer an ecosystem for microorganisms from diverse phylogenetic domains and phyla as well as viruses and viroids. Bacteria, fungi, and oomycetes represent the most important microbial core of plants. These microorganisms intimately interact with plant cells and tissues with different levels of dependence, ranging from mutualism to pathogenicity. Some of these microorganisms cause harm by deploying diverse strategies to attack plants and impair plant growth and reproduction, while others improve plant growth through different physiological activities that may have profound effects on plants’ growth and/or health. They are also able to compete with pathogens for nutrients and niches or exert antagonism through antimicrobial compounds. These beneficial microbes are also able to interfere with pathogen signals or trigger plant host immunity. The papers in this Special Issue illustrate some aspects related to the implication of microorganisms in plant life, including their various roles and their characterization and identification.

The rapid growth of the world population generates considerable needs in terms of quantity and quality of food products. At the same time, many pathogens and pests attack cultivated plants, which, until now, have been controlled mainly by synthetic pesticides. The environmental risks generated by the use of these chemicals, their harmful impact on human health and the desire of the population for healthier food free of pesticides have led to the emergence of the concept of biological control of plant diseases. However, biocontrol is facing a quadruple paradigm: (i) feeding around 9 billion humans by 2050, (ii) decreasing pesticide use, (iii) affording an efficient protection against plant pathogens and (iv) facing some skepticism as regard to the efficacity of biocontrol. All these points are discussed in the comprehensive review of Lahlali et al. [1]. Generalization of the use of chemical pesticides and the distrust of populations towards GMOs for the protection of plants have generated a major interest in methods for the biocontrol of plant diseases in the respect of the environment. Biocontrol not only integrates phytosanitary aspects but also the socio-economic context (legislative and marketing aspects). The antagonistic biocontrol agents (BCAs) that are under use belong to over 440 species including bacterial species, namely (but in a non-exhaustive way), *Bacillus*, *Burkholderia* spp., *Pseudomonas* and *Trichoderma*, as well as fungal species such as *Aspergillus, Beauveria*, *Fusarium*, *Penicillium* and *Phoma* spp. Most of the BCAs come from the rhizosphere community, commonly named the rhizosphere microbiome. These microorganisms include beneficial bacteria (plant-growth-promoting rhizobacteria) able to promote not only plant growth but also plant disease resistance through the production of numerous antifungal compounds (secondary metabolites and antibiotics), fungi and several yeasts. Authors then explored the possible modes of action performed by the BCAs, which are divided into i) direct modes of action such as antibiosis, parasitism, reduction in pathogen virulence and disease pressure, production of lytic enzymes and antimicrobial compounds, formation of biofilms as well as competition for nutrients and space and ii) indirect modes of action (induced resistance and priming implying numerous signaling pathways, phytohormones, defense compounds such as phytoalexins and PR proteins). Application of biocontrol in the field is facing numerous constraints. The first of them is the long and laborious screening of potentially useful strains from the laboratory to the field. The second is the limited number of registered bio-fungicide products. Finally, BCA survival is confronted by environmental factors (e.g., climate changes, sun light exposure) and limited shelf life. Importantly, the article provides a very complete overview of the trade names of the biopesticides currently under use, their target disease/pathogen as well as the manufacturers and the distributors which produce and sell them. The review ends with a discussion about the factors which affect success and failure of biocontrol use in the field.

Endophytic microorganisms (bacteria, fungi, algae) display multiple beneficial effects on their host plants such as an increase in the resistance to pathogens as well as tolerance to abiotic stresses (cold, dryness); the promotion of plant growth and increase in crop yields through the production of hormones; and an improvement in nutrients uptake, etc. These latter aspects and the appropriate characterization of endophytes in plants are described in the following five papers [2,3,4,5,6].

*Pseudowintera colorata* (horopito) is a medicinal plant from the subalpine areas of New Zealand used in traditional Maori medicine to cure various ailments such as skin infections, fever, gonorrhea and toothache. If the endophytic bacterial community and that of actinobacteria have already been studied in that plant, no report has been published regarding its endophytic fungal community to date. The article of Purushotham et al. [2] provides the first report on the fungal endophytic flora of *P. colorata*. PCR-denaturing gradient gel electrophoresis showed the relationship between the different endophytic fungal communities in this plant and the type of organ, as well as the plant’s age. A total of 200 endophytic fungi were recovered from sterilized organs (leaves, stems and roots) and grouped into 50 morphotypes whose identification was performed by sequencing of the non-coding DNA internal transcribed spacer (ITS) of ribosomes. Interestingly, among the 50 endophytic fungi tested, some of them displayed a high inhibitory activity against phytopathogenic fungi, while others exhibit a significant antagonistic action on human opportunistic pathogens. Moreover, the endophytic fungi showed a positive effect on *P. colorata* growth regarding plant height and the number of internodes. This work thus reports for the first time the characterization of the endophytic fungal communities in *P. colorata*, as well as its potential benefits in terms of resistance to disease and plant growth.

Leafless epiphytic orchids are plants of extreme habitats where periods of drought and high exposure to ultra-violet light prevail. These plants, which have very reduced leaves, have set up a system of aerial roots developing a photosynthetic activity and a C4 (Crassulacean acid) metabolism typical of plants of arid climates. These roots also present a particular anatomical structure made up of a multi-layered spongy tissue, the velamen conducive to association with many microorganisms. On the other hand, there are no data existing on bacterial associations taking place at the level of these aerial roots. In the work of Tsavkelova et al. [3], a characterization of the cyanobacteria colonizing the aerial roots of two leafless orchids, *Chiloschista parishii* and *Microcoelia moreauae,* is presented. DGGE analysis and subsequent 16S rDNA sequencing revealed that the major cyanobacteria were nitrogen-fixing cyanobacteria belonging to three dominating orders, Oscillatoriales, Synechococcales and Nostocales. The cyanobacterial morphotypes formed biofilms covering the entire surface of the aerial roots of *C. parishii* or simple cavities at the root surface in *M. moreauae*, but studies failed to demonstrate any cyanobacterial localization inside velamen cells. Changes in these cyanobacteria communities were observed according to environmental conditions. Namely, under deprived nitrogen supply, two cyanobacterial populations prevailed belonging to the genera *Komarekiella* and *Leptolyngbya*, demonstrating an adaptation to environmental changes. This work thus constitutes the first sound description of the cyanobacterial root communities of leafless epiphytic orchids.

Endophytic microorganisms are particularly useful to the plants they live with, especially for biological nitrogen fixation. In this area, nitrogen-fixing rhizobacteria are of great interest to decrease the use of N-fertilizers in the fields. N_2_-fixation is reliant on nitrogenase activity, which is related to microbial growth, the latter being linked with plant-borne carbon and the level of photosynthate availability. In their study, Waller et al. [4] reported on the influence of various nitrogen inputs on growth and nitrogen fixation capability of the endophytic rhizobacterium, *Herbaspirillum seropedicae*, in maize. When the plant was deprived of nitrates (1 m*M*), there was a resultant increase in nitrogenase activity along with an enhanced demand for plant carbon as compared to high nitrogen concentrations (10 m*M*). By applying ^11^C radio-tracing following administration of ^11^CO_2_, authors were able to put in evidence that, under low nitrogen, there was a reduction in CO_2_ fixation and a concomitant decrease in translocation of the photosynthates (as determined by measuring the level of plant-borne ^11^C) to the roots and thus limitation in bacterial growth. The study also suggested that maintaining a high nitrogen fixation activity in a low nitrogen environment might be balanced by providing a carbon source to the soil, which has less deleterious effects than synthetic nitrogen supply.

The article of Hwang et al. [5] illustrates the role of an endophytic rhizobacterium, *Burkholderia seminalis* 869T2, isolated from the roots of vetiver grass (*Chrysopogon zizanioides*) as a promoting agent for plant growth (vegetative organs) of Arabidopsis, ching chiang pak choi, pack choi, lettuce and Chinese amaranth, in addition to having a positive influence on flowering in hot pepper and okra. *B. seminalis* possibly exerted its promoting effect on plant growth through the production of indole acetic acid, as evidenced by hormone measurements in bacterial cultures in the presence of tryptophan. It was also reasoned that the plant-growth-promoting activity of *B. seminalis* 869T2 could be mediated by its ability to synthesize siderophores as a chelating agent for iron Fe^3+^ cations and its aptitude for phosphate solubilization. This study thus strongly supports the potential of this *Burkholderia* strain as a bioinoculant for applications in agriculture.

*Botrytis cinerea* is a necrotrophic fungal pathogen, which possesses hundreds of different host plants, among which are tomato, strawberry, sunflower, cucumber, grapevine, etc. The main curative methods to fight this pathogen remain the use of pesticides. The development of *Botrytis* strains resistant to these chemical treatments, the cost of these treatments, their environmental consequences and the consumer demand for pesticide-free food, make the search for alternative methods of combating this disease a necessity. The work of the Ait-Barka group [6] described the isolation and the screening of soil beneficial bacteria for the biological control of *B. cinerea*. Potential antagonistic bacteria were collected from soils from healthy Moroccan vineyards and from those severely attacked by this fungus. Of the 42 pure cultures obtained, two isolates, namely, S3 and S6, exhibited a significant reduction in *Botrytis* mycelial growth (35 and > 20%, respectively) on PDA plates. Both strains displayed an inhibition of *Botrytis* growth of around 50% at 72 hpi on whole potted-grapevine. These observations were confirmed by experiments realized in in vitro plantlets inoculated with the strains S3 and S6 regarding *Botrytis* infection. Moreover, a photosynthetic analysis study revealed that both strains prevent or moderate PSII damage caused by *B. cinerea* attacks on the bacterized in vitro plantlets. Strains S3 and S6 were identified after 16S rRNA gene sequence analysis and draft genome sequence comparisons, as *Bacillus velezensis* (99% average nucleotide identity, ANI) and *Enterobacter cloacae* (98% ANI). Potential applications of these antagonistic bacteria for *B. cinerea* control in various environments were also discussed.

As said before, microorganisms are not always beneficial to the plants they interact with. Indeed, many of them are harmful, with deleterious effects on their hosts. Four papers in this Special Issue describe pathogen interactions (bacteria, fungi, oomycetes and parasitic nematodes) with various plants [7,8,9,10]. The work of Havrlentová et al. [7] reported the consequences of artificial *Fusarium* infection on the quality of oat grain (*Avena sativa*). Inoculation was realized with two *Fusarium* species, *F. graminearum* and *F. culmorum*, responsible for Fusarium head blight, a cereal fungal disease of wheat, barley, oat, rye and triticale. Experimental data first showed a decrease in the starch content of oat grains, which could be explained by the use of this polysaccharide by the pathogen. In contrast, no conclusive trends were drawn regarding the lipid contents of the infected grains compared to controls. However, except oleic acid, the concentrations of all other main fatty acids of the oat grain (linoleic and palmitic acids) were found to increase following artificial inoculation. Unsurprisingly, a significant rise in the mycotoxin deoxynivalenol was also observed after fungal inoculation as a sign of the infection process. Consequences of the changes in the analytical composition of oat grains resulting from fungal infection for the food industry were further discussed.

Factual observations made by the group of Lahlali [8] in the Saïss plain of Morocco during the 2017–2018 growing season reported several marked symptoms on apple and pear trees characteristic of the apple and pear decline diseases. After collection of 50 samples from five different locations in the areas of Fez-Meknes, the causal agent of the disease symptoms observed was an oomycete and not related to an infection by *Phytophthora* or *Pythium* species. A total of 35 oomycete isolates recovered from the different sites underwent both morphological and molecular identifications. The morphological traits (shape and color, modes of growth and sporulation) of the colonies grown on PDA medium were typical of oomycetes. Further characterization using *Cox II* gene sequencing, as well as phylogenetic tree analyses, concluded that the pathogen responsible for the symptoms observed was *Phytopythium vexans*. Greenhouse pathogenicity tests carried out with all the oomycete isolates revealed a symptom similarity with the trees found in the field. Observations also reported a higher disease prevalence in sandy-clay soils, likely due to a better mobility of the zoospores in this type of soils facilitating their way to the roots. The data obtained in this pioneering study have allowed the identification of a new causal agent of apple and pear decline and afford the bases for the management of this disease in apple and pear orchards.

Stubborn is a citrus disease present worldwide in dry or semi-arid regions and rather rare under cool climates. This disease is difficult to identify, as its symptomatology is not very different from that of other plant injuries caused by biotic and abiotic stresses. Sagouti et al. [9] reported, in a comprehensive review, the recurrent problems caused by this disease in all regions where citrus grows and especially in those of the Mediterranean rim. Citrus stubborn disease is caused by *Spiroplasma citri*, a phloem-restricted bacterial pathogen of the Spiroplasmataceae family. From a taxonomy point of view, *S. citri* belongs to the *Bacteria* of the *Firmicutes* phylum and the class of *Mollicutes*. Its genome size is 1780 Kbp and presents in its structure a circular chromosome along with plasmids and viral sequences, the biological significance of the latter being unknown. Gene sequence analysis revealed that the strains of *S. citri* are closely related but not similar. Stubborn symptomatology is dependent on high temperatures, and symptom severity seems to be linked to the bacterial titer rather than to the strain virulence. Citrus stubborn disease is vectorized by numerous leafhopper species, as well as by grafting or collection of buds from infected plants. Leafhoppers are able to transfer the bacteria to weeds and the transmission to citrus is ensured by the infected weeds. The review moves next to the different methods enabling detection of the disease (isolation of *S. citri* in culture media, ELISA, PCR, nested PCR, real-time PCR, IC-PCR, RFLP, targeting the spiralin gene and biological indexing). Strategies for disease control may include introduction of *S. citri*-free buds into citrus orchards; the use of antibiotics, insecticides and fungicides revealing problems of resistant strains; and prospects around the engineering of genes encoding for antimicrobial peptides (AMPs). Authors concluded on the need to further understand the epidemiology of *S. citri* and to identify the mechanisms underlying the spread of this pathogenic bacterium worldwide.

Turkey is one of the top 10 wheat-producing countries in the world, with an average annual production of 20 million tons. Plant-parasitic nematodes represent the main parasites of cereal crops being responsible of yield losses of about 10%. The objective of the work of Keçici et al. [10] was to afford data on the diversity, the identification and the molecular phylogeny of parasitic nematode populations of the cereals of the provinces of Sakarya, a region of intensive cereal production. Plant-parasitic nematodes belonging to 13 major taxa were found in most of the collected soil samples (92%) from this area. The cys nematode *Heterodera filipjevi* accounted for 24% of the nematodes identified from the soil samples. In contrast, the nematodes were not present in case of crop rotations with non-cereal crops, underlying the fact that there is a strong association between these parasites and cereals and, beyond, monoculture systems in agriculture. This was confirmed by determining the Shannon biodiversity index, which revealed a moderate migratory ability of the nematodes within wheat fields. Based on these data, field practice recommendations (use of crop rotations, wheat breeding for high-resistance to cys nematodes) were made.

To conclude this editorial, we will further develop some aspects regarding the mechanisms through which BCAs may generate a response from the host plant, as well as commercialization challenges that the use of BCAs is facing. Effects of BCAs on plant defense stimulation and plant growth promotion result from very diverse mechanisms including the induction of signaling pathways namely involving salicylic acid, jasmonic acid, ethylene [11,12] as well as elicitation of induced systemic resistance by rhizobacteria [13]. All these reactions end by the synthesis of numerous compounds implied in the biochemical defense responses of plants, such as PR-proteins, structural barriers, phytoalexins and defense-related enzymes such as phenylalanine ammonia lyase and chitinases. Some of these defense responses are related with both the systemic acquired resistance and the induced systemic resistance or priming concept [14]. BCAs’ plant-growth-promoting effects, on the other hand, are to be connected to the increased production of several plant growth regulators such as auxins, gibberellins, cytokinins and abscisic acid. In contrast, BCAs can also counteract the action of ethylene, a stress marker, through inhibition of its production via the 1-aminocyclopropane-1-carboxylic acid (ACC) synthase.

The commercialization of BCAs faces many challenges. First, the market value of BCAs represents less than 5% for the entire phytosanitary sector [15]. This is largely due to a limited number of registered formulations, though this number is rapidly increasing, as 64% of the BCAs’ species approved by the United States Environmental Protection Agency have been registered for the last ten years [16]. The proportion of BCAs strains employed for pest management in the field only accounts for 1% of the protection methods used in agriculture [16]. This should be compared to the percentage of fungicides used, which accounts for 15% of the total chemicals employed [17]. Finally, it can be noted that the manufacturers/producers of biopesticide active agents belong to a very limited number of countries, mainly the USA, followed by a few in Europe (Spain, Belgium, The Netherlands and Austria) [1].

To conclude with, future prospects in biocontrol will concern, on one hand, deepening our fundamental knowledge on the mechanisms taking place upon the interaction of BCAs and their host plants. From a practical point of view, it is of prime importance to pursue the selection of new organisms for biocontrol at the lab and field scale as well as to understand and manage the factors compromising BCAs’ efficacy, such as the risk factors which can facilitate the emergence of plant pathogen strains resistant to BCAs. This encompasses the management of environmental factors, physiological and genetic improvement of the mechanisms of biocontrol, the use of microorganism combinations and new formulations of the biopesticide active agents.

The papers presented in this Special Issue concern different aspects of the interrelations between plants and microorganisms, and authors hope this will convince young researchers to work on this field of research.
